# The genome sequence of the Heath Knot-horn, *Apomyelois bistriatella* (Hulst, 1887)

**DOI:** 10.12688/wellcomeopenres.19306.1

**Published:** 2023-04-12

**Authors:** James Hammond, University of Oxford and Wytham Woods Genome Acquisition Lab, Darwin Tree of Life Barcoding collective, Wellcome Sanger Institute Tree of Life programme, Wellcome Sanger Institute Scientific Operations: DNA Pipelines collective, Tree of Life Core Informatics collective, Darwin Tree of Life Consortium

**Affiliations:** 1 Department of Biology, University of Oxford, Oxford, England, OX2 8QJ, UK

**Keywords:** Apomyelois bistriatella, Heath Knot-horn, genome sequence, chromosomal, Lepidoptera

## Abstract

We present a genome assembly from an individual female *Apomyelois bistriatella* (the Heath Knot-horn; Arthropoda; Insecta; Lepidoptera; Pyralidae). The genome sequence is 389.6 megabases in span. Most of the assembly is scaffolded into 32 chromosomal pseudomolecules, including the Z and W sex chromosomes. The mitochondrial genome has also been assembled and is 15.2 kilobases in length.

## Species taxonomy

Eukaryota; Metazoa; Ecdysozoa; Arthropoda; Hexapoda; Insecta; Pterygota; Neoptera; Endopterygota; Lepidoptera; Glossata; Ditrysia; Pyraloidea; Pyralidae; Phycitinae; *Apomyelois*; *Apomyelois bistriatella* (Hulst, 1887) (NCBI:txid1666458).

## Background

*Apomyelois bistriatella* (Hulst, 1887) is a moth of the Pyralidae family. It has a circumpolar distribution, being found across North America and northern Eurasia, ranging from the British Isles in the west to Hokkaido in the east ( GBIF Secretariat, 2022; Neunzig, 1990). In Europe it is represented by the subspecies *neophanes* ( Goater *et al.*, 1986; Neunzig, 1990). The species has a scattered distribution in the British Isles, favouring heathy locations ( Goater *et al.*, 1986; Parsons & Davis, 2018).

Within the British Isles, larvae have been recorded feeding within the fungus *Daldinia concentrica*, growing on burnt Gorse ( *Ulex europeaus*) or young birches ( *Betula*) ( Goater *et al.*, 1986), however in North America the species is also known to feed within *Hypoxylon* fungi, growing on recently killed oak ( *Quercus*) or poplar ( *Populus*) ( Neunzig, 1990). Larvae feed between August and October, after which the larva burrows into dead wood or fungus and overwinters ( Goater *et al.*, 1986; Parsons & Davis, 2018). Pupation occurs during May ( Parsons & Davis, 2018). Adults fly at night between May and September, resting by day on tree trunks with the head raised away from the body and the tips of the forewings pressed against the trunk ( Goater *et al.*, 1986; Parsons & Davis, 2018). Colonies of this species are ephemeral, and can move around according to the availability of young birch and burnt gorse ( Goater *et al.*, 1986).

The genome of *Apomyelois bistriatella* was sequenced as part of the Darwin Tree of Life Project, a collaborative effort to sequence all named eukaryotic species in the Atlantic Archipelago of Britain and Ireland. Here we present a chromosomally complete genome sequence for *Apomyelois bistriatella*, based on one female specimen of the subspecies *neophanes* from Wytham Woods, Oxfordshire, UK.

## Genome sequence report

**Figure 1.**
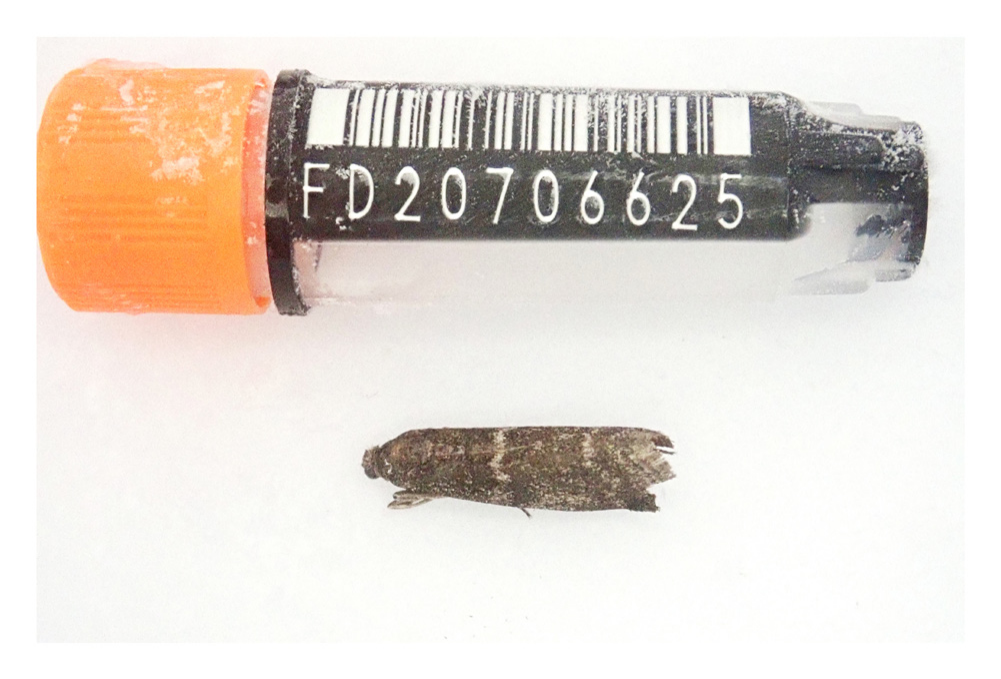
Photograph of the *Apomyelois bistriatella* (ilApoBist1) specimen used for genome sequencing.

The genome was sequenced from one female *Apomyelois bistriatella* ( Figure 1) collected from Wytham Woods, Oxfordshire, UK (latitude 51.77, longitude –1.34). A total of 70-fold coverage in Pacific Biosciences single-molecule HiFi long reads was generated. Primary assembly contigs were scaffolded with chromosome conformation Hi-C data. Manual assembly curation corrected nine missing or mis-joins and removed one haplotypic duplication, reducing the scaffold number by 13.16%.

**Figure 2.**
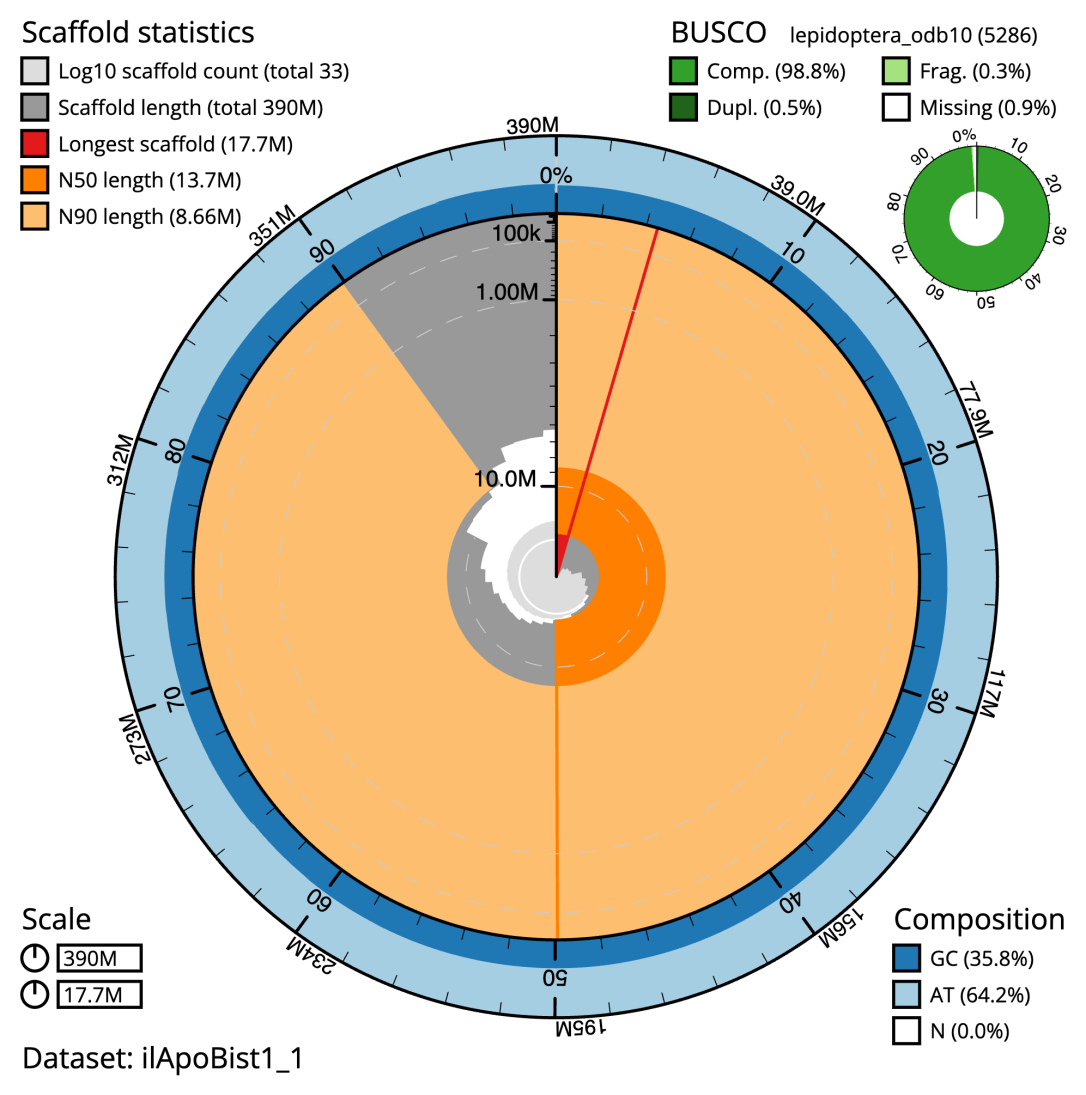
Genome assembly of *Apomyelois bistriatella*, ilApoBist1.1: metrics. The BlobToolKit Snailplot shows N50 metrics and BUSCO gene completeness. The main plot is divided into 1,000 size-ordered bins around the circumference with each bin representing 0.1% of the 389,571,851 bp assembly. The distribution of scaffold lengths is shown in dark grey with the plot radius scaled to the longest scaffold present in the assembly (17,721,000 bp, shown in red). Orange and pale-orange arcs show the N50 and N90 scaffold lengths (13,669,180 and 8,655,929 bp), respectively. The pale grey spiral shows the cumulative scaffold count on a log scale with white scale lines showing successive orders of magnitude. The blue and pale-blue area around the outside of the plot shows the distribution of GC, AT and N percentages in the same bins as the inner plot. A summary of complete, fragmented, duplicated and missing BUSCO genes in the lepidoptera_odb10 set is shown in the top right. An interactive version of this figure is available at https://blobtoolkit.genomehubs.org/view/ilApoBist1.1/dataset/ilApoBist1_1/snail.

The final assembly has a total length of 389.61 Mb in 33 sequence scaffolds with a scaffold N50 of 13.7 Mb ( Table 1). Most (99.99%) of the assembly sequence was assigned to 32 chromosomal-level scaffolds, representing 30 autosomes, and the Z and W sex chromosomes. Chromosome-scale scaffolds confirmed by the Hi-C data are named in order of size ( Figure 2– Figure 5; Table 2). While not fully phased, the assembly deposited is of one haplotype. Contigs corresponding to the second haplotype have also been deposited. The mitochondrial genome was also assembled and can be found as a contig within the multifasta file of the genome submission.

**Table 1.** Genome data for *Apomyelois bistriatella*, ilApoBist1.1.

Project accession data		
Assembly identifier	ilApoBist1.1	
Species	*Apomyelois bistriatella*	
Specimen	ilApoBist1	
NCBI taxonomy ID	1666458	
BioProject	PRJEB55343	
BioSample ID	SAMEA10978762	
Isolate information	ilApoBist1, female: whole organism (genome sequencing and Hi-C scaffolding)	
Assembly metrics *		*Benchmark*
Consensus quality (QV)	68.2	*≥ 50*
*k*-mer completeness	100%	*≥ 95%*
BUSCO **	C:98.8%[S:98.3%,D:0.5%], F:0.3%,M:0.9%,n:5,286	*C ≥ 95%*
Percentage of assembly mapped to chromosomes	99.99%	*≥ 95%*
Sex chromosomes	Z and W	*localised homologous pairs*
Organelles	Mitochondrial genome assembled	*complete single alleles*
Raw data accessions		
PacificBiosciences SEQUEL II	ERR10077564	
Hi-C Illumina	ERR10084071	
Genome assembly		
Assembly accession	GCA_947044815.1	
*Accession of alternate haplotype*	GCA_947044225.1	
Span (Mb)	389.6	
Number of contigs	41	
Contig N50 length (Mb)	13.4	
Number of scaffolds	33	
Scaffold N50 length (Mb)	13.7	
Longest scaffold (Mb)	17.7	

*Assembly metric benchmarks are adapted from column VGP-2020 of “Table 1: Proposed standards and metrics for defining genome assembly quality” from ( Rhie *et al.*, 2021). **BUSCO scores based on the lepidoptera_odb10 BUSCO set using v5.3.2. C = complete [S = single copy, D = duplicated], F = fragmented, M = missing, n = number of orthologues in comparison. A full set of BUSCO scores is available at https://blobtoolkit.genomehubs.org/view/ilApoBist1.1/dataset/ilApoBist1_1/busco.

**Figure 3.**
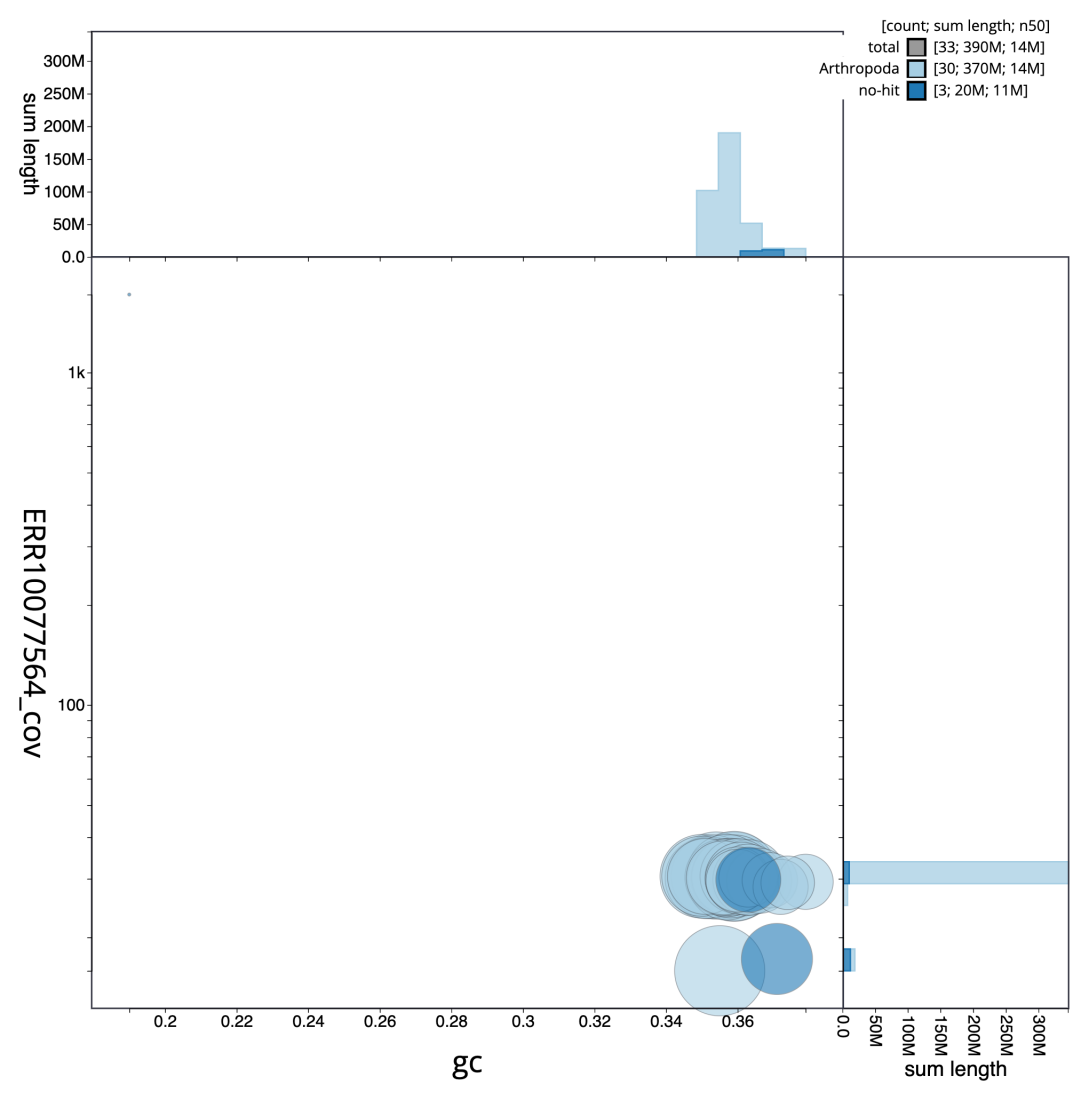
Genome assembly of *Apomyelois bistriatella*, ilApoBist1.1: BlobToolKit GC-coverage plot. Scaffolds are coloured by phylum. Circles are sized in proportion to scaffold length. Histograms show the distribution of scaffold length sum along each axis. An interactive version of this figure is available at https://blobtoolkit.genomehubs.org/view/ilApoBist1.1/dataset/ilApoBist1_1/blob.

**Figure 4.**
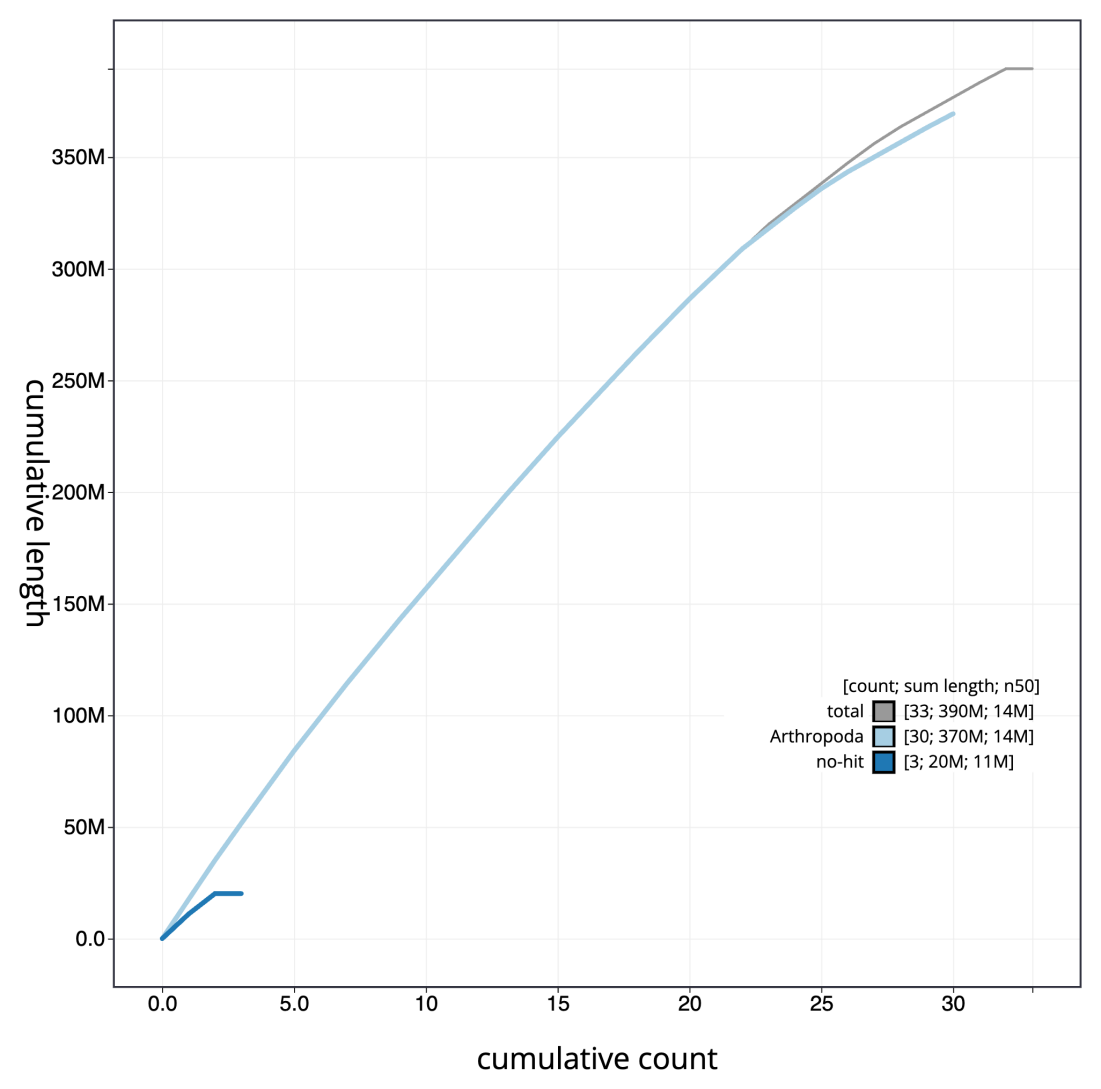
Genome assembly of *Apomyelois bistriatella*, ilApoBist1.1: BlobToolKit cumulative sequence plot. The grey line shows cumulative length for all scaffolds. Coloured lines show cumulative lengths of scaffolds assigned to each phylum using the buscogenes taxrule. An interactive version of this figure is available at https://blobtoolkit.genomehubs.org/view/ilApoBist1.1/dataset/ilApoBist1_1/cumulative.

**Figure 5.**
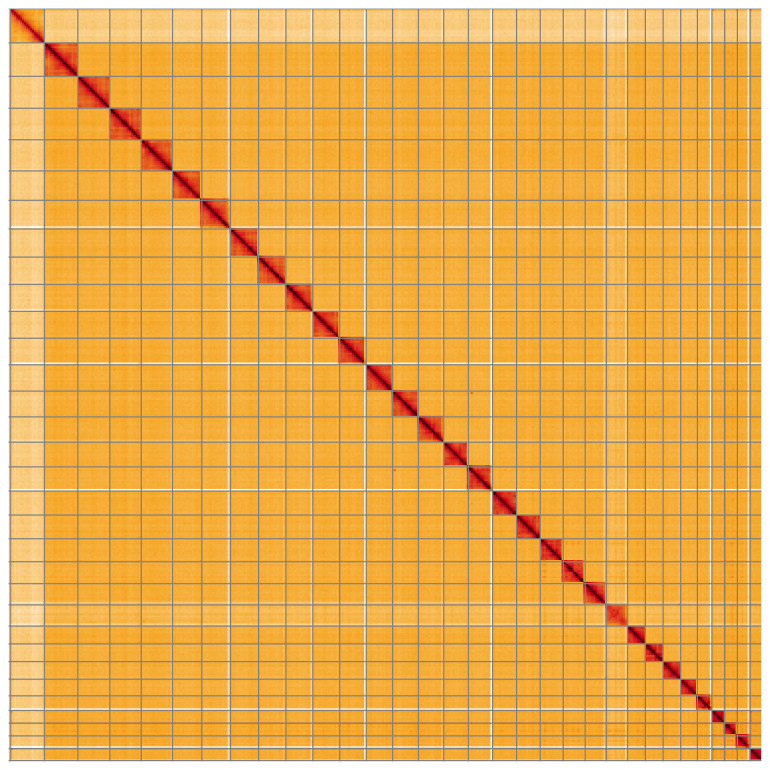
Genome assembly of *Apomyelois bistriatella*, ilApoBist1.1: Hi-C contact map of the ilApoBist1.1 assembly, visualised using HiGlass. Chromosomes are shown in order of size from left to right and top to bottom. An interactive version of this figure may be viewed at https://genome-note-higlass.tol.sanger.ac.uk/l/?d=Olwsrw1tR7mMGnnJQB8skA.

**Table 2.** Chromosomal pseudomolecules in the genome assembly of *Apomyelois bistriatella*, ilApoBist1.

INSDC accession	Chromosome	Size (Mb)	GC%
OX345688.1	1	17.33	35.9
OX345689.1	2	16.57	35.4
OX345690.1	3	16.29	35.9
OX345691.1	4	16.16	35.9
OX345692.1	5	15.22	35
OX345693.1	6	14.87	35.3
OX345694.1	7	14.54	35.2
OX345695.1	8	14.14	35.1
OX345696.1	9	13.96	35
OX345697.1	10	13.95	35.7
OX345698.1	11	13.72	35.7
OX345699.1	12	13.67	35.8
OX345700.1	13	13.35	35.7
OX345701.1	14	13.12	35.8
OX345702.1	15	12.78	35.6
OX345703.1	16	12.51	35.1
OX345704.1	17	12.46	36.3
OX345705.1	18	12.27	36
OX345706.1	19	11.9	35.6
OX345707.1	20	11.38	36.1
OX345708.1	21	10.99	36.3
OX345710.1	22	9.21	36.6
OX345711.1	23	9.16	36.3
OX345712.1	24	9.1	36
OX345713.1	25	8.66	36
OX345714.1	26	7.52	36.3
OX345715.1	27	6.64	36.9
OX345716.1	28	6.59	37.9
OX345717.1	29	6.58	37.2
OX345718.1	30	6.22	37.4
OX345709.1	W	10.99	37.1
OX345687.1	Z	17.72	35.5
OX345719.1	MT	0.02	19.3

The estimated Quality Value (QV) of the final assembly is 68.2 with *k*-mer completeness of 100%, and the assembly has a BUSCO v5.3.2 completeness of 98.8%% (single = 98.3%, duplicated = 0.5%), using the lepidoptera_odb10 reference set ( *n* = 5,286).

## Methods

### Sample acquisition and nucleic acid extraction

A female *Apomyelois bistriatella* specimen (ilApoBist1) was collected from Wytham Woods, Oxfordshire (biological vice-county: Berkshire), UK (latitude 51.77, longitude –1.34) on 30 June 2021. The specimen was taken from woodland habitat by James Hammond (University of Oxford) using a light trap. The specimen was identified by the collector and preserved on dry ice. 

The ilApoBist1 sample was weighed and dissected on dry ice with tissue set aside for Hi-C sequencing. Whole organism tissue was disrupted using a Nippi Powermasher fitted with a BioMasher pestle. DNA was extracted from whole organism tissue of ilApoBist1 at the Wellcome Sanger Institute (WSI) Scientific Operations core using the Qiagen MagAttract HMW DNA kit, according to the manufacturer’s instructions.

### Sequencing

Pacific Biosciences HiFi circular consensus DNA sequencing libraries were constructed according to the manufacturers’ instructions. DNA sequencing was performed by the Scientific Operations core at the WSI on Pacific Biosciences SEQUEL II (HiFi) instrument. Hi-C data were also generated from tissue of ilApoBist1 using the Arima v2 kit and sequenced on the Illumina NovaSeq 6000 instrument.

### Genome assembly, curation and evaluation

Assembly was carried out with Hifiasm ( Cheng *et al.*, 2021) and haplotypic duplication was identified and removed with purge_dups ( Guan *et al.*, 2020). The assembly was then scaffolded with Hi-C data ( Rao *et al.*, 2014) using YaHS ( Zhou *et al.*, 2023). The assembly was checked for contamination as described previously ( Howe *et al.*, 2021). Manual curation was performed using HiGlass ( Kerpedjiev *et al.*, 2018) and Pretext ( Harry, 2022). The mitochondrial genome was assembled using MitoHiFi ( Uliano-Silva *et al.*, 2022), which performed annotation using MitoFinder ( Allio *et al.*, 2020). To evaluate the assembly, MerquryFK was used to estimate consensus quality (QV) scores and *k*-mer completeness ( Rhie *et al.*, 2020). The genome was analysed and BUSCO scores ( Manni *et al.*, 2021; Simão *et al.*, 2015) were calculated within the BlobToolKit environment ( Challis *et al.*, 2020). Table 3 contains a list of software tool versions and sources.

**Table 3.** Software tools: versions and sources.

Software tool	Version	Source
BlobToolKit	4.0.7	https://github.com/blobtoolkit/blobtoolkit
BUSCO	5.3.2	https://gitlab.com/ezlab/busco
Hifiasm	0.16.1-r375	https://github.com/chhylp123/hifiasm
HiGlass	1.11.6	https://github.com/higlass/higlass
Merqury	MerquryFK	https://github.com/thegenemyers/MERQURY.FK
MitoHiFi	2	https://github.com/marcelauliano/MitoHiFi
PretextView	0.2	https://github.com/wtsi-hpag/PretextView
purge_dups	1.2.3	https://github.com/dfguan/purge_dups

### Ethics and compliance issues

The materials that have contributed to this genome note have been supplied by a Darwin Tree of Life Partner. The submission of materials by a Darwin Tree of Life Partner is subject to the Darwin Tree of Life Project Sampling Code of Practice. By agreeing with and signing up to the Sampling Code of Practice, the Darwin Tree of Life Partner agrees they will meet the legal and ethical requirements and standards set out within this document in respect of all samples acquired for, and supplied to, the Darwin Tree of Life Project. All efforts are undertaken to minimise the suffering of animals used for sequencing. Each transfer of samples is further undertaken according to a Research Collaboration Agreement or Material Transfer Agreement entered into by the Darwin Tree of Life Partner, Genome Research Limited (operating as the Wellcome Sanger Institute), and in some circumstances other Darwin Tree of Life collaborators.

## Data Availability

European Nucleotide Archive: *Apomyelois bistriatella.* Accession number PRJEB55343, https://identifiers.org/ena.embl/PRJEB55343. ( Wellcome Sanger Institute, 2022) The genome sequence is released openly for reuse. The *Apomyelois bistriatella* genome sequencing initiative is part of the Darwin Tree of Life (DToL) project. All raw sequence data and the assembly have been deposited in INSDC databases. The genome will be annotated using available RNA-Seq data and presented through the Ensembl pipeline at the European Bioinformatics Institute. Raw data and assembly accession identifiers are reported in Table 1.

## Author information

Members of the University of Oxford and Wytham Woods Genome Acquisition Lab are listed here: https://doi.org/10.5281/zenodo.4789928.

Members of the Darwin Tree of Life Barcoding collective are listed here: https://doi.org/10.5281/zenodo.4893703.

Members of the Wellcome Sanger Institute Tree of Life programme are listed here: https://doi.org/10.5281/zenodo.4783585.

Members of Wellcome Sanger Institute Scientific Operations: DNA Pipelines collective are listed here: https://doi.org/10.5281/zenodo.4790455.

Members of the Tree of Life Core Informatics collective are listed here: https://doi.org/10.5281/zenodo.5013541.

Members of the Darwin Tree of Life Consortium are listed here: https://doi.org/10.5281/zenodo.4783558.
