# Supplementary material for: The genome sequence of the Heath Knot-horn, Apomyelois bistriatella (Hulst, 1887)
Source: Wellcome Open Res. 2023 Apr 12;8:164. [Version 1] doi: 10.12688/wellcomeopenres.19306.1 (PMC10905011; doi:10.12688/wellcomeopenres.19306.1)
Supplement: Peer review history

# Reviewer report: Reviewer response for version 1

**DOI:** 10.21956/wellcomeopenres.21389.r73589

**Published:** 2024-02-16

**Authors:** Andrew Mongue

**Affiliations:** 1 Department of Entomology and Nematology, University of Florida, Gainesville, Florida, USA

This manuscript reports another gold-standard insect genome, as is the norm for those generated by the Tree of Life Project. The data and tools are well documented and available. My only question is around the sex chromosomes.

 The author reports the Z and W chromosomes but does not give details on how they were identified from the whole genome asssembly. The sex chromosomes should have half the coverage of the autosomes, but how were the Z and W distinguished from each other? On the basis of size or homology to other species with known sex chromosomes?

 Even one brief sentence on this would be enough to clarify.

Are sufficient details of methods and materials provided to allow replication by others?

Yes

Is the rationale for creating the dataset(s) clearly described?

Yes

Are the datasets clearly presented in a useable and accessible format?

Yes

Are the protocols appropriate and is the work technically sound?

Yes

Reviewer Expertise:

evolutionary genomics, sex chromosomes, Lepidoptera

I confirm that I have read this submission and believe that I have an appropriate level of expertise to confirm that it is of an acceptable scientific standard.

# Reviewer report: Reviewer response for version 1

**DOI:** 10.21956/wellcomeopenres.21389.r73596

**Published:** 2024-02-16

**Authors:** Kay Lucek

**Affiliations:** 1 Biodiversity Genomics Laboratory, University of Neuchâtel, Neuchâtel, Switzerland

The authors present the chromosome level genome assembly of the Heath Knot-horn, Apomyelois bistriatella. The assembly consists of 32 chromosomes including both sex chromosomes. The assembly is highly complete as revealed by the high BUSCO score but not fully phased. Sequencing and genome assembly follow the current state of the art and use established methods. Overall, the presented assembly will be of great value to study genome architecture as well as genome size evolution in Lepidoptera.

 It states that ”the genome will be annotated using available RNA-Seq data”, however, according to Table 1 this data has not yet been deposited.

Are sufficient details of methods and materials provided to allow replication by others?

Yes

Is the rationale for creating the dataset(s) clearly described?

Yes

Are the datasets clearly presented in a useable and accessible format?

Partly

Are the protocols appropriate and is the work technically sound?

Yes

Reviewer Expertise:

Speciation genomics

I confirm that I have read this submission and believe that I have an appropriate level of expertise to confirm that it is of an acceptable scientific standard.
